# 2-Chloro-3-nitro­pyridine

**DOI:** 10.1107/S1600536810011955

**Published:** 2010-04-02

**Authors:** Seik Weng Ng

**Affiliations:** a Department of Chemistry, University of Malaya, 50603 Kuala Lumpur, Malaysia

## Abstract

In the title compound, C_5_H_3_ClN_2_O_2_, the nitro group is twisted by 38.5 (2)° with respect to the pyridine ring. In the crystal, adjacent mol­ecules are linked by non-classical C—H⋯N and C—H⋯O hydrogen bonds, forming a layer motif.

## Related literature

For the crystal structure of isostructural 2-iodo-3-nitro­pyridine, see: Mao & Chen (2009 ▶). For the crystal structure of 2-chloro-5-nitro­pyridine, see: Ng (2010 ▶).
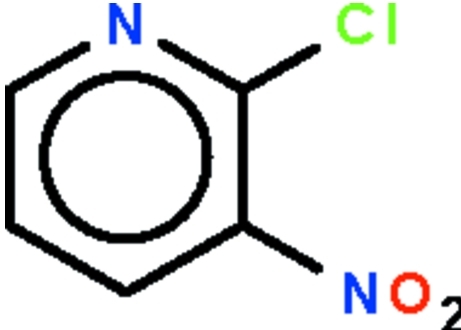


## Experimental

### 

#### Crystal data

C_5_H_3_ClN_2_O_2_
*M* *_r_* = 158.54
Monoclinic, 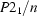

*a* = 7.613 (1) Å
*b* = 12.232 (2) Å
*c* = 7.716 (1) Å
β = 118.485 (2)°
*V* = 631.5 (2) Å^3^
*Z* = 4
Mo *K*α radiation
μ = 0.53 mm^−1^
*T* = 293 K
0.30 × 0.20 × 0.05 mm

#### Data collection

Bruker SMART APEX diffractometer
Absorption correction: multi-scan (*SADABS*; Sheldrick, 1996 ▶) *T*_min_ = 0.771, *T*_max_ = 0.862
5889 measured reflections
1445 independent reflections
1061 reflections with *I* > 2σ(*I*)
*R*_int_ = 0.040

#### Refinement

*R*[*F* ^2^ > 2σ(*F* ^2^)] = 0.037
*wR*(*F* ^2^) = 0.108
*S* = 1.02
1445 reflections
103 parameters
3 restraints
All H-atom parameters refined
Δρ_max_ = 0.22 e Å^−3^
Δρ_min_ = −0.29 e Å^−3^

### 

Data collection: *APEX2* (Bruker, 2009 ▶); cell refinement: *SAINT* (Bruker, 2009 ▶); data reduction: *SAINT*; program(s) used to solve structure: *SHELXS97* (Sheldrick, 2008 ▶); program(s) used to refine structure: *SHELXL97* (Sheldrick, 2008 ▶); molecular graphics: *X-SEED* (Barbour, 2001 ▶); software used to prepare material for publication: *publCIF* (Westrip, 2010 ▶).

## Supplementary Material

Crystal structure: contains datablocks global, I. DOI: 10.1107/S1600536810011955/im2183sup1.cif

Structure factors: contains datablocks I. DOI: 10.1107/S1600536810011955/im2183Isup2.hkl

Additional supplementary materials:  crystallographic information; 3D view; checkCIF report

## Figures and Tables

**Table 1:** Hydrogen-bond geometry (Å, °)

*D*—H⋯*A*	*D*—H	H⋯*A*	*D*⋯*A*	*D*—H⋯*A*
C3—H3⋯N1^i^	0.93 (1)	2.53 (1)	3.430 (3)	166 (2)
C4—H4⋯O1^ii^	0.93 (1)	2.64 (2)	3.327 (3)	132 (2)

Symmetry codes: (i) 
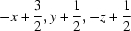
; (ii) 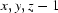.

**Fig. 1.**
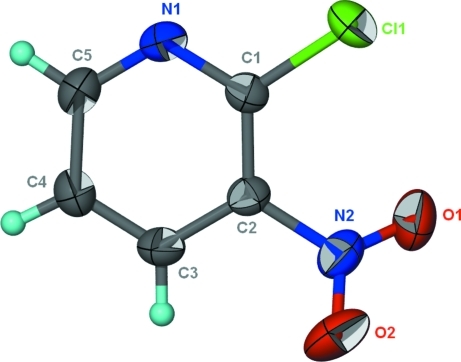
Thermal ellipsoid plot (Barbour, 2001) of C5H3ClNO2 at the 50% probability level; hydrogen atoms are drawn as spheres of arbitrary radius.

**Fig. 2.**
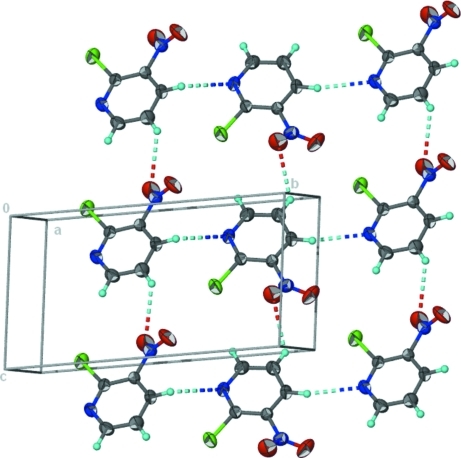
Non-classical hydrogen-bonded layer motif.

## supplementary crystallographic information

### Comment

According to a recent report on the crystal structure of 2-chloro-5-nitropyridine the respective molecule is planar (maximum r.m.s. deviation of non-hydrogen atoms is 0.090 Å). This molecule has the electron withdrawing substituents *para* to each other. The substituents interact through a short Cl···O contact of 3.068 (4) Å to generate a chain motif (Ng, 2010).

In the title compound 2-chloro-3-nitropyridine with the nitro group *ortho* to the chlorine substituent (Scheme I, Fig. 1), a similar Cl···O contact is also observed but the nitro group is twisted to avoid repulsion. Adjacent molecules are linked by non-classical C–H···N and C–H···O hydrogen bonds to form a layer motif (Fig. 2, Table 1). The C–H···N interaction is almost linear (Table 1).

2-Chloro-3-nitropyridine is isostructural with the iodo analog. In the iodo compound, the I···O contact is necessarily longer (Mao & Chen, 2009).

### Experimental

2-Chloro-3-nitropyridine was obtained from the Aldrich Chemical Company, and was recrystallized from ethyl acetate.

### Refinement

Carbon bound H-atoms were located in a difference Fourier map. They were refined with a distance restraint of C–H 0.93±0.01 Å; their temperature factors were refined without constraints.

### Crystal data

**Table:**

C_5_H_3_ClN_2_O_2_	*F*(000) = 320
*M**_r_* = 158.54	*D*_x_ = 1.668 Mg m^−^^3^
Monoclinic, *P*2_1_/*n*	Mo *K*α radiation, λ = 0.71073 Å
Hall symbol: -P 2yn	Cell parameters from 1393 reflections
*a* = 7.613 (1) Å	θ = 3.3–24.8°
*b* = 12.232 (2) Å	µ = 0.53 mm^−^^1^
*c* = 7.716 (1) Å	*T* = 293 K
β = 118.485 (2)°	Block, faint yellow
*V* = 631.5 (2) Å^3^	0.30 × 0.20 × 0.05 mm
*Z* = 4	

### Data collection

**Table:**

Bruker SMART APEX diffractometer	1445 independent reflections
Radiation source: fine-focus sealed tube	1061 reflections with *I* > 2σ(*I*)
graphite	*R*_int_ = 0.040
ω scans	θ_max_ = 27.5°, θ_min_ = 3.1°
Absorption correction: multi-scan (*SADABS*; Sheldrick, 1996)	*h* = −9→9
*T*_min_ = 0.771, *T*_max_ = 0.862	*k* = −15→15
5889 measured reflections	*l* = −9→10

### Refinement

**Table:**

Refinement on *F*^2^	Primary atom site location: structure-invariant direct methods
Least-squares matrix: full	Secondary atom site location: difference Fourier map
*R*[*F*^2^ > 2σ(*F*^2^)] = 0.037	Hydrogen site location: inferred from neighbouring sites
*wR*(*F*^2^) = 0.108	All H-atom parameters refined
*S* = 1.02	*w* = 1/[σ^2^(*F*_o_^2^) + (0.0539*P*)^2^ + 0.1169*P*] where *P* = (*F*_o_^2^ + 2*F*_c_^2^)/3
1445 reflections	(Δ/σ)_max_ = 0.001
103 parameters	Δρ_max_ = 0.22 e Å^−^^3^
3 restraints	Δρ_min_ = −0.28 e Å^−^^3^

### Fractional atomic coordinates and isotropic or equivalent isotropic displacement parameters (Å^2^)

**Table:**

	*x*	*y*	*z*	*U*_iso_*/*U*_eq_	
Cl1	0.67770 (9)	0.67094 (4)	0.54558 (9)	0.0583 (2)	
O1	0.5971 (3)	0.89254 (15)	0.6322 (3)	0.0793 (6)	
O2	0.8242 (3)	1.00241 (15)	0.6464 (3)	0.0804 (6)	
N1	0.6852 (3)	0.71189 (13)	0.2214 (3)	0.0471 (4)	
N2	0.7123 (3)	0.92525 (15)	0.5760 (3)	0.0514 (5)	
C1	0.6891 (3)	0.75880 (14)	0.3767 (3)	0.0381 (4)	
C2	0.7109 (3)	0.87120 (14)	0.4062 (3)	0.0375 (4)	
C3	0.7322 (3)	0.93592 (16)	0.2719 (3)	0.0468 (5)	
C4	0.7252 (4)	0.88674 (18)	0.1090 (3)	0.0522 (5)	
C5	0.7011 (3)	0.77541 (19)	0.0896 (3)	0.0516 (5)	
H3	0.751 (3)	1.0104 (9)	0.294 (3)	0.060 (7)*	
H4	0.740 (3)	0.9261 (17)	0.013 (3)	0.061 (7)*	
H5	0.693 (3)	0.7407 (18)	−0.021 (2)	0.060 (7)*	

### Atomic displacement parameters (Å^2^)

**Table:**

	*U*^11^	*U*^22^	*U*^33^	*U*^12^	*U*^13^	*U*^23^
Cl1	0.0742 (4)	0.0455 (3)	0.0605 (4)	−0.0024 (2)	0.0366 (3)	0.0121 (2)
O1	0.1151 (16)	0.0782 (12)	0.0759 (13)	0.0077 (11)	0.0709 (13)	−0.0011 (9)
O2	0.0855 (13)	0.0709 (11)	0.0755 (12)	−0.0105 (10)	0.0308 (11)	−0.0382 (10)
N1	0.0558 (11)	0.0367 (8)	0.0490 (10)	−0.0028 (7)	0.0252 (9)	−0.0068 (7)
N2	0.0605 (12)	0.0499 (10)	0.0427 (10)	0.0115 (8)	0.0236 (9)	−0.0031 (8)
C1	0.0370 (10)	0.0354 (9)	0.0399 (10)	0.0002 (7)	0.0168 (8)	0.0023 (7)
C2	0.0385 (10)	0.0340 (8)	0.0378 (10)	0.0031 (7)	0.0164 (8)	−0.0014 (7)
C3	0.0585 (13)	0.0324 (9)	0.0497 (12)	−0.0014 (8)	0.0260 (10)	−0.0009 (8)
C4	0.0671 (14)	0.0491 (12)	0.0507 (13)	−0.0024 (10)	0.0366 (11)	0.0038 (9)
C5	0.0640 (14)	0.0520 (12)	0.0447 (12)	−0.0029 (10)	0.0307 (11)	−0.0083 (9)

### Geometric parameters (Å, °)

**Table:**

Cl1—C1	1.7226 (18)	C2—C3	1.374 (3)
O1—N2	1.217 (2)	C3—C4	1.371 (3)
O2—N2	1.213 (2)	C3—H3	0.925 (9)
N1—C1	1.317 (2)	C4—C5	1.373 (3)
N1—C5	1.330 (3)	C4—H4	0.930 (10)
N2—C2	1.462 (2)	C5—H5	0.929 (10)
C1—C2	1.391 (3)		
			
C1—N1—C5	118.07 (17)	C4—C3—C2	118.15 (18)
O2—N2—O1	124.60 (19)	C4—C3—H3	122.2 (15)
O2—N2—C2	117.20 (19)	C2—C3—H3	119.7 (15)
O1—N2—C2	118.15 (18)	C3—C4—C5	118.67 (19)
N1—C1—C2	121.95 (16)	C3—C4—H4	122.2 (15)
N1—C1—Cl1	115.43 (14)	C5—C4—H4	119.2 (15)
C2—C1—Cl1	122.55 (14)	N1—C5—C4	123.54 (18)
C3—C2—C1	119.58 (17)	N1—C5—H5	116.5 (15)
C3—C2—N2	117.52 (16)	C4—C5—H5	120.0 (15)
C1—C2—N2	122.90 (17)		
			
C5—N1—C1—C2	−0.5 (3)	O2—N2—C2—C1	143.2 (2)
C5—N1—C1—Cl1	−177.56 (15)	O1—N2—C2—C1	−39.3 (3)
N1—C1—C2—C3	−1.1 (3)	C1—C2—C3—C4	1.9 (3)
Cl1—C1—C2—C3	175.72 (15)	N2—C2—C3—C4	−178.09 (19)
N1—C1—C2—N2	178.96 (18)	C2—C3—C4—C5	−1.2 (3)
Cl1—C1—C2—N2	−4.2 (3)	C1—N1—C5—C4	1.3 (3)
O2—N2—C2—C3	−36.8 (3)	C3—C4—C5—N1	−0.4 (4)
O1—N2—C2—C3	140.7 (2)		

### Hydrogen-bond geometry (Å, °)

**Table:**

*D*—H···*A*	*D*—H	H···*A*	*D*···*A*	*D*—H···*A*
C3—H3···N1^i^	0.93 (1)	2.53 (1)	3.430 (3)	166 (2)
C4—H4···O1^ii^	0.93 (1)	2.64 (2)	3.327 (3)	132 (2)

Symmetry codes: (i) −*x*+3/2, *y*+1/2, −*z*+1/2; (ii) *x*, *y*, *z*−1.
